# When the sum isn’t the whole - The vascular status score and its components in hypertension identification: The African PREDICT study

**DOI:** 10.1038/s41371-026-01156-3

**Published:** 2026-05-09

**Authors:** Marizelle Vermeulen, Wayne Smith, Yolandi Breet, Annemarie Wentzel

**Affiliations:** 1 Hypertension in Africa Research Team (HART), North-West University, Potchefstroom, South Africa; 2 South African Medical Research Council Unit for Hypertension and Cardiovascular Disease, North-West University, Potchefstroom, South Africa

**Keywords:** Risk factors, Hypertension

## Abstract

The vascular status score (VSS) is an emerging composite index, developed to investigate early vascular changes by integrating pulse wave velocity (PWV), augmentation index (AIx), and carotid intima-media thickness (cIMT). Although prior studies have linked the VSS to inflammatory and metabolic dysfunction, its association with hypertension remains unexplored. This study compared the ability of the VSS and its individual components in identifying hypertension, regardless of phenotype, among young South African adults. We included 1,019 participants aged 20 - 30 years from a community-based cohort. Office and ambulatory blood pressures were measured, and hypertension phenotypes were defined according to the 2024 ESC guidelines. Carotid-femoral PWV, Aix, and cIMT were assessed and combined to calculate the composite VSS. Receiver operating characteristic (ROC) analyses determined optimal cut points for VSS and its components relative to normotensive and hypertensive classifications, and odds ratios were adjusted for relevant covariates. Among the indices examined, only PWV demonstrated consistent sensitivity and specificity across hypertension phenotypes. While a VSS above 4 modestly increased the odds of masked, sustained, and white-coat hypertension (odds ratios ranging from 1.74 to 2.35, all *P* < 0.05), only PWV accurately identified masked and sustained hypertension (odds ratios 2.25–2.70, all *P* < 0.01). Neither AIx nor cIMT achieved comparable diagnostic utility. Despite the promise of a composite vascular index, PWV independently outperformed the VSS and its other components in detecting hypertension in young adults. These findings suggest that PWV may be the most reliable individual biomarker for early hypertension identification in young adults.

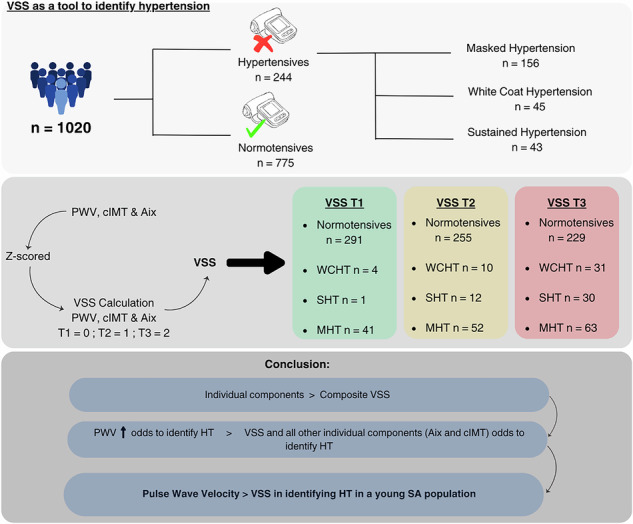

## Introduction

Cardiovascular disease (CVD) remains the leading cause of premature death globally and the primary contributor to mortality in South Africa [1, 2]. Hypertension (HT) is the most prevalent and modifiable CVD risk factor, and is increasingly detected among young South African adults, many of whom remain undiagnosed until vascular dysfunction or organ damage becomes evident [3–5]. Early and accurate identification of vascular alterations preceding overt hypertension is therefore a critical clinical priority [6].

Although widely used, conventional risk models such as the Framingham Risk Score and Pooled Cohort Equations primarily estimate long-term atherosclerotic disease risk and often underperform in African populations. Their limited generalizability and predictive accuracy underscores the need for alternative or complementary tools that directly assess vascular health and capture early pathophysiological changes underlying hypertension [7], as well as the identification of novel, population-specific tools, particularly those capable of identifying the most prevalent and modifiable cardiovascular risk factor, hypertension, in young, seemingly healthy individuals.

Hypertension is recognized as a heterogeneous condition comprising distinct phenotypes: sustained (SHT), masked (MHT), and white-coat hypertension (WCHT), each defined by different patterns of office and ambulatory blood pressure (BP) [8]. These phenotypes vary not only in BP presentation but also in their vascular profiles and cardiovascular outcomes: sustained and masked hypertension are associated with markedly higher vascular risk, while white-coat hypertension carries a comparatively modest but progressive vascular burden. Such heterogeneity highlights the importance of vascular markers and/or biomarkers for comprehensive risk stratification and identification [9].

The heterogeneity of hypertension phenotypes underscores the need for improved risk stratification strategies tailored to this demographic. Non-invasive vascular biomarkers such as pulse wave velocity (PWV), augmentation index (AIx), and carotid intima-media thickness (cIMT) individually reflect key pathophysiological aspects of arterial stiffness, wave reflection, and subclinical atherosclerosis, respectively [10–16]. Yet each marker captures only a single dimension of vascular health, and their combined interpretation may yield greater diagnostic and phenotypic insight.

A population-based study in a Scandinavian cohort has proposed a composite Vascular Status Score (VSS) that combines PWV, AIx, and cIMT measures into one, distinct measure [17–19]. Petterson-Pablo and co-workers reported that the VSS might detect functional alterations in the vasculature preceding metabolic changes [17–19]. This may suggest the possibility of early, non-invasive identification of hypertension without the need for invasive, traditional cardiometabolic biomarkers, such as cholesterol, glucose and inflammatory measures. Early evidence from this Scandinavian cohort suggests that the VSS may detect subclinical vascular dysfunction preceding metabolic or inflammatory changes, positioning it as a promising, non-invasive measure for early vascular risk detection, and may be useful in hypertension identification, regardless of phenotype. Given the unique genetic, environmental, and socioeconomic factors influencing cardiovascular risk profiles in a diverse South African population, the evaluation of such a composite measure is both timely and clinically relevant. However, it remains unclear whether the composite VSS can better identify hypertension and its phenotypes than its individual components, in a young South African population and do so more accurately than its individual components.

Therefore, this study aimed to evaluate the diagnostic utility of the composite VSS relative to PWV, AIx, and cIMT in identifying hypertension phenotypes among young South African adults (20–30 years old).

## Methods

### Study population

This study forms part of the African PRospective study on the Early Detection and Identification of Cardiovascular Disease and HyperTension (African-PREDICT) [20]. The recruitment of participants occurred in and around the Potchefstroom area, North-West province of South Africa. Young, apparently healthy adults aged 20 to 30 years were screened to determine whether they met the inclusion criteria of the study. The specific exclusion criteria included a mean systolic blood pressure of 140 mm Hg and/or a diastolic pressure of 90 mmHg or higher, as well as human immunodeficiency virus (HIV) infection, a prior diagnosis of any chronic disease, pregnancy, or breastfeeding [20, 21].

Our study included the previously collected cross-sectional data from the baseline phase of the African-PREDICT study (*n* = 1202). Participants with incomplete or missing data for office blood pressure (BP), 24-hour blood pressure, PWV, AIx, cIMT, and individuals with an ABPM inflation rate of less than 70% were excluded from this study (*n* = 183). Thus N = 1,019 participants were included in subsequent analysis. This study complies with the Declaration of Helsinki (2008), the South African Department of Health Research In human participants Document (revised in 2024) and the Health Research Ethics Committee of North-West University (NWU-00001-12-A1). The African-PREDICT study is registered in a clinical trials registry (NCT03292094). All procedures were thoroughly explained to the participants and written informed consent was obtained before any measurements were performed [20].

### Questionnaires and general demographics

Participants completed a general demographic and health questionnaire with the assistance of a trained researcher. The following information was collected: age, sex, ethnicity, smoking habits, alcohol intake, and medication use. Contraceptive use was considered during the statistical analysis due to the large number of young women in this population using contraceptives [22], as it has a known influence on vascular compliance and BP [23]. The socioeconomic status (SES) of a participant was determined based on three categories from the general health questionnaire: skill level, education, and household income. Each category was assigned a specific number of points, and the total points were used to classify the participants into low, middle, or high SES and a continuous SES score [21]. Importantly, SES was considered as a covariate, as SES influences vascular health, particularly blood pressure, through pathways like stress, poor diet, obesity, and limited healthcare access, with unique patterns in South Africa due to inequality and epidemiological transition. Lower SES often correlates with higher hypertension risk, mediated by bio-behavioural factors [24].

### Anthropometric measurements

#### Body composition

All anthropometric measurements were done in triplicate by following the guidelines specified by ISAK (International Society for the Advancement of Kinanthropometry) [25] by a trained research assistant. Body height (m) (SECA 213 Stadiometer (SECA, Hamburg, Germany)), body weight (kg) (SECA 813 Electronic scale (SECA, Hamburg, Germany)) and waist circumference (WC) (cm) (Lufkin steel anthropometric tape (W606PM; Lufkin, Apex, USA)). The body mass index (BMI) was calculated as: (weight (kg) / height (m2)) [20].

### Cardiovascular measurements

#### Office blood pressure

Blood pressure (Dinamap® Procare 100 device (GE Medical Systems, Milwaukee, USA)) with GE Critikon latex-free Dura-Cuffs (medium and large). Participants were in an upright, seated position. Measurements were taken in duplicate on each alternating arm at 5-minute intervals. Systolic (SBP) and diastolic blood pressure (DBP) were measured, and the mean arterial pressure (MAP) and pulse pressure (PP) were subsequently calculated [20]. The mean of the two readings was used for all subsequent analyses.

#### 24-hour ambulatory blood pressure

The CardioXplore devices (Meditech, Budapest, Hungary) were used for the collection of 24-h BP measurements and programmed to measure blood pressure at 30-minute intervals during the day (from 06:00 to 22:00) and every hour during the night (from 22:00 to 06:00), unsuccessful measurements were repeated automatically. The ABPM was fitted to the participant’s non-dominant arm with an appropriate size cuff. Only participants with >70% valid 24-h BP measurements (inflation rate) were included in the analyses to ensure sufficient data extraction.

#### Defining BP phenotypes

Although participants were screened to be normotensive a week to 10 days earlier to their follow-up visit, advanced BP and clinical measures indicated that undiagnosed HT was evident in multiple cases. The overall hypertensive ABPM BP status was determined as follows: 24 h ABPM BP status (SBP ≥ 130 mm Hg; DBP ≥ 80), ABPM night BP status (SBP ≥ 120; DBP ≥ 70 mm Hg) and ABPM day BP status (SBP ≥ 135; DBP ≥ 85) [8]. Whereafter participants were classified with *sustained hypertension* (SHT) if they had a hypertensive office BP (SBP ≥ 140; DBP ≥ 90) and an overall ABPM hypertensive status (as discussed above) resulting in an ABPM SBP ≥ 130 and ABPM DBP ≥ 80. *White-coat hypertension* (WCHT) if participants had normal 24 h ABPM (SBP ≤ 129; DBP ≤ 79) but hypertensive office BP SBP ≥ 140; DBP ≥ 90). *Masked hypertension* (MHT) if the participant had normal office blood pressure (SBP ≤ 139; DBP ≤ 89) but hypertensive 24 h ABPM measurement [8]. For this study, we also grouped individuals in the *all-cause hypertension* group if they showed any BP measurement within the hypertensive ranges for both the office BP and ABPM measurements. Thus, if they had an office BP measurement and/or an ABPM measurement within the hypertensive ranges, they were defined as all-cause HT.

#### Pulse wave velocity and augmentation index

The Sphygmocor® XCEL device (AtCor Medical Pty. Ltd., Sydney, Australia) was used to perform pulse wave analysis and measure carotid-femoral PWV with the participants in a supine position. Pulse wave analysis was performed with a brachial cuff placed on the right upper arm, with central systolic BP and the AIx estimated by a general transfer function [20].

For the carotid-femoral PWV measurement, a femoral arterial waveform was captured by placing the cuff around the right thigh of the participant while the right carotid arterial waveform was captured simultaneously via applanation tonometry. The direct distance from the carotid pulse to the upper femoral cuff was measured (transit-distance method), after which 80% of this distance was used to represent the pulse wave travel distance [26]. The measurement was taken in duplicate and if the measurements differed by more than 0.5 m/s they were repeated [20]. The average of the two cfPWV measurements that were closest to each other was used in our analyses.

#### Carotid intima-media thickness

The General Electric Vivid E9, GE Vingmed Ultrasound A/S in Horten, Norway, was operated by a trained sonographer. A registered clinical technician then analysed the images obtained. Images of both the left and right common cIMT was measured offline using a dedicated software (Carotid Analyzer for Research v.6, Medical Imaging Applications, Coralville, Iowa, USA) [27]. Three optimal angles for each side of the common carotid artery were documented, and the average thickness for both sides was calculated accordingly [20]. In this study, the average of the left and right near and far wall measurements were used.

#### Compilation of the VSS

The data from the PWV, Aix and cIMT were transformed into z-scores, thus a z-score for each variable. These z-scores were then categorized into the VSS based on the tertiles for the PWV, Aix and cIMT measurements respectively [19]. Each category was assigned a number with 0 corresponding to the lowest category and adding 1 for each additional category. The VSS was divided into tertiles to be able to note the change in vascular profile that occurs across changes in the arterial tree, and the presence of BP phenotypes in each tertile. The score range is thus 0–3 for median-based scores and 0–6 for tertile-based scores, with the third tertile indicating the most compromised vascular profile (with high PWV, AIx, and cIMT) [19].

### Blood and/or serum sampling and biochemical analysis

Basic serum analyses were done to determine the levels of C-reactive protein (high sensitivity), γ-glutamyl transferase (GGT), triglycerides, high-density lipoprotein cholesterol (HDL-C), low-density lipoprotein cholesterol (LDL-C) and total cholesterol (TC) by using the Cobas Integra 400plus (Roche, Basel Switzerland). Glucose levels were determined in sodium fluoride plasma samples using the Cobas Integra 400plus (Roche, Basel Switzerland). Serum Cotinine levels were determined by applying the Chemiluminescence method to the Immulite (Siemens, Erlangen, Germany). Serum Insulin levels were determined by means of the Electrochemiluminescence method on the E411 (Roche, Basel Switzerland). The homeostatic model assessment of insulin resistance (HOMA-IR), indicating sub-clinical insulin resistance, was determined by using the following calculation method: $\frac{{Glucose}\left(\frac{{mmol}}{L}\right)\times {Insulin}\left(\right.\frac{\mu U}{{l}^{-8}}}{22.5}$ [20].

### Statistical analysis

All statistical analyses were performed by using IBM® SPSS® Statistics version 28 software (IBM Corporation; Armonk, New York, USA. Descriptive comparisons were expressed across tertiles of the VSS to enable stratification of subclinical vascular changes that may precede overt hypertension and to maintain balanced baseline group sizes. Categorical variables were summarized as frequencies and percentages and compared using the Chi-square test. Continuous variables were presented as mean ± standard deviation for normally distributed data or as median (interquartile range) for skewed data, with distributional normality assessed using the Shapiro-Wilk test. Between-group comparisons were performed using one-way analysis of variance (ANOVA) or the Kruskal-Wallis test, as appropriate. Bonferroni post hoc corrections were applied to account for multiple comparisons.

Nonparametric receiver operating characteristic (ROC) analyses were used to determine optimal cut points for the composite VSS and its components (PWV, AIx, and cIMT) in identifying each hypertension phenotype. Adjusted odds ratios (ORs) and 95% confidence intervals (CIs) were derived from logistic regression models to evaluate the likelihood of detecting hypertension at each estimated cut point. Covariates entered into all regression models included age, sex, ethnicity, SES, WC, MAP (supine, SphygmoCor), hs-CRP, total cholesterol, and self-reported smoking and alcohol use. As the primary vascular measures (PWV, AIx, cIMT and the combined VSS), are influenced by demographic, socioeconomic, and cardiometabolic factors, and controlling for these parameters ensures more accurate effect estimates [28]. Adjusting logistic regression PWV, AIX, cIMT, and VSS is standard to isolate their unique associations while minimizing confounding bias. These vascular measures are influenced by demographic, socioeconomic, and cardiometabolic factors, and controlling for them ensures more accurate effect estimates. Age strongly predicts arterial stiffening (PWV/AIX) and cIMT via collagen deposition and elastin loss; sex differences arise from hormonal effects (estrogen protects pre-menopause), while ethnicity captures genetic/environmental variations, like higher PWV in Black South Africans [29–31]. These are non-modifiable but potent confounders. As mentioned, we also adjusted for SES, as lower socioeconomic status correlates with higher hypertension/PWV through stress, poor diet, and healthcare access disparities, prominent in South Africa [24, 32]. Anthropometric/hemodynamic (WC, MAP supine SphygmoCor) were included, as WC reflects visceral adiposity driving endothelial dysfunction/cIMT; mean arterial pressure directly affects shear stress and PWV, requiring adjustment to avoid overestimation. Inflammatory/lipid (hs-CRP, total cholesterol), specifically hs-CRP indicated low-grade inflammation promoting atherosclerosis/cIMT, while cholesterol contributes to plaque formation, confounding vascular scores, therefore justifying baseline adjustment [33, 34]. Lifestyle factors especially smoking and alcohol use were included as smoking accelerates PWV/cIMT via oxidative stress/vasoconstriction, and alcohol has dose-dependent vascular effects, both needing control for residual confounding [35, 36].

Sensitivity analysis was conducted to assess the influence of contraceptive use among women (n = 226) on model outcomes; inclusion of this variable showed no significant effect, and these participants were therefore retained in all analyses. Two-tailed tests were applied throughout, with statistical significance set at *P* < 0.05.

## Results

The basic characteristics of the study population stratified across tertiles of the VSS are presented in Table 1 The mean age of the total group was 24.50 ± 3.11 years, with individuals in tertile 3 being the oldest (P for trend <0.001). Tertile 3 was composed of mostly men (54.7%) of Black ethnicity (52.4%) (P for trend <0.001 for both). The average body mass index (BMI), waist circumference (WC), BP measurements, total cholesterol, triglycerides, insulin, GGT, and HOMA-IR all increased across tertiles of the VSS (P for trend <0.001). A positive trend was observed across VSS tertiles, with higher VSS values corresponding to increased BMI, total cholesterol, and triglyceride levels. Furthermore, glucose, LDL-C, and HDL-C levels did not differ significantly across VSS tertiles in our study population. The prevalence of hypertension, across all phenotypes (WCHT, MHT SHT and All-cause HT), increased across tertiles of the VSS, with the highest number of participants within these phenotypes in tertile 3 of the VSS.

**Table 1:** Basic Characteristics of the study population stratified across tertiles of the VSS.

	Total Group n = 1019	VSS Tertile 1 n = 337	VSS Tertile 2 n = 329	VSS Tertile 3 n = 353	P-Value
**Demographic and Lifestyle Parameters**					
Age (years)	24.50 ± 3.11	23.52 ± 2.90^a***^	24.53 ± 3.11^b***^	25.42 ± 3.04^c***^	<0.001
Sex (men, %)	490 (48.0)	136 (40.2)^♦^ (13.3)^◊^	161 (48.9)^♦^ (15.8)^◊^	193 (54.7)^♦^ (18.9) ^◊^	<0.001
Ethnicity (Black, n %)	475 (46.6)	127 (37.6)^♦^ (12.5)^◊^	163 (49.5)^♦^ (16.0)^◊^	185 (52.4)^♦^ (18.1)^◊^	<0.001
Body mass index (Kg/m^2^)	23.89 (20.96; 27.43)	22.96 (20.46; 26.09)^a***^	24.21 (21.31; 27.86)	24.63 (21.40; 28.15)^c***^	<0.001
Waist circumference (cm)	77.50 (70.5; 86.50)	74.80 (69.18; 83.58)^a***^	78.50 (71.13; 86.65)	79.50 (71.95; 90.35)^c***^	<0.001
Alcohol use, n (%)	569 (55.8)	169 (50.4)^♦^ (16.7)^◊^	184 (56.1)^♦^ (18.2)^◊^	216 (61.7)^♦^ (21.3)^◊^	0.012
Smoking status, yes n (%)	236 (23.2)	63 (18.7)^♦^ (6.2)^◊^	81 (24.6)^♦^ (7.9)^◊^	92 (26.1)^♦^ (9.0)^◊^	0.054
Contraceptive use, n (%)	226 (22.2)	83 (24.6)^♦^ (8.1)^◊^	69 (21.0)^♦^ (6.8)^◊^	74 (21.0)^♦^ (7.3)^◊^	0.430
Physical activity (TEE) kCal/day n = 828	2217 (1961; 2523)	2125 (1921; 2430)	2257 (2021; 2561)	2247 (1985; 2539)	0.315
Socioeconomic status					0.475
Low n (%)	383 (37.5)	134 (39.8)^♦^ (13.2)^◊^	121 (36.8)^♦^ (11.9)^◊^	22 (6.3)^♦^ (12.6)^◊^	0.048
Middle n (%)	306 (30.0)	107 (31.8)^♦^ (10.5)^◊^	96 (29.2)^♦^ (9.4)^◊^	33 (9.2)^♦^ (10.1)^◊^	0.096
High n (%)	330 (32.4)	96 (28.5)^♦^ (9.4)^◊^	112 (34.0)^♦^ (11.0)^◊^	122 (34.6)^♦^ (12.0)^◊^	0.138
**Blood pressure measurements**					
24 h SBP (mmHg)	116 (110; 123)	114 (107; 120)^a***^	117 (111; 124)	118 (112;125)^c***^	<0.001
24 h DBP (mmHg)	68 (65; 72)	66 (63; 70)^a***^	68 (65; 72)^b***^	71 (67; 75)^c***^	<0.001
24 h PP (mmHg)	47 (43; 53)	47 (43; 53)	48 (44; 53)^b**^	47 (42; 52)	0.010
24 h MAP (mmHg)	88 (83; 92)	85 (81; 89)^a***^	88 (84; 92)^b***^	90 (85; 94)^c***^	<0.001
Office SBP (mmHg)	118 (110; 127)	113 (107; 122)^a***^	118 (111; 126)^b***^	121 (113; 131)^c***^	<0.001
Office DBP (mmHg)	78 (74; 83)	75 (71; 79)^a***^	78 (74; 82)^b***^	82 (77; 87)^c***^	<0.001
Office PP (mmHg)	39 (33; 45)	38 (32; 44)	39 (33; 45)	39 (33; 45)	0.270
Clinic MAP (mmHg)	93 (88; 100)	91 (85; 95)^a***^	94 (88; 100)^b***^	97 (91; 103)^c***^	<0.001
SygmoCor MAP (mmHg)	86 (82; 92)	82 (78; 87)^a***^	87 (82; 91)^b***^	92 (87; 98)^c***^	<0.001
Heart rate (Beats/min)	64 (57; 71)	62 (56; 69)^a**^	64 (57; 71)^b*^	66 (59; 74)^c***^	<0.001
**BP Phenotypes**					
Normotensive, n (%)	775 (76.1)	291 (86.4)^♦^ (28.6)^◊^	255 (77.5)^♦^ (25.0)^◊^	229 (64.9)^♦^ (22.5)^◊^	<0.001
White-coat, n (%)	45 (4.4)	4 (1.2)^♦^ (0.4)^◊^	10 (3.0)^♦^ (1.0)^◊^	31 (8.8)^♦^ (3.0)^◊^	<0.001
Masked, n (%)	156 (15.3)	41 (12.2)^♦^ (4.0)^◊^	52 (15.8)^♦^ (5.1)^◊^	63 (17.8)^♦^ (6.2)^◊^	<0.001
Sustained hypertensive, n (%)	43 (4.2)	1 (0.3)^♦^ (0.1)^◊^	12 (3.6)^♦^ (1.2)^◊^	30 (8.5)^♦^ (2.9)^◊^	<0.001
All Cause Hypertensive status, n (%)^●^	244 (23.9)	46 (13.6)^♦^ (4.5)^◊^	74 (22.5)^♦^ (7.3)^◊^	124 (35.1)^♦^ (12.2)^◊^	<0.001
**Macrovascular Measurements**					
PWV (m/s)▪	6.38 (6.28; 6.47)	5.78 (5.69; 5.87)^a***^	6.36 (6.28; 6.45)^b***^	6.94 (6.85; 7.03)^c***^	<0.001
Aix (%)	4.0 (−4.0; 11.5)	−3.5 (−9.5; 1.0)^a***^	3.5 (−6.0; 11.75)^b***^	11.0 (6.5; 17.5)^c***^	<0.001
cIMT (mm)	0.44 (0.07)	0.41 (0.04)	0.45 (0.08)	0.51 (0.07) ^c***^	0.001
Total VSS	4 (3; 5)	3 (2; 3)^a***^	4 (4; 4)^b***^	5 (5; 6)^c***^	<0.001
**Biochemical markers**					
Total cholesterol (mmol/l)	3.66 (2.91; 4.55)	3.52 (2.82; 4.39)	3.45 (2.83; 4.38)^b***^	3.93 (3.06; 4.86)^c***^	<0.001
HDL-C (mmol/l)	1.12 (0.84; 1.40)	1.14 (0.83; 1.40)	1.09 (0.83; 1.37)	1.14 (0.86; 1.45)	0.330
LDL-C (mmol/l)	2.29 (1.74; 3.02)	2.27 (1.70; 2.92)	2.24 (1.69; 2.92)^b**^	2.42 (1.84; 3.30)^c*^	0.002
Triglycerides (mmol/l)	0.69 (0.50; 0.99)	0.63 (0.47; 0.91)	0.71 (0.50; 0.94)^b***^	0.77 (0.54; 1.16)^c***^	<0.001
C-reactive protein (mg/l)	0.81 (0.29; 2.33)	0.62 (0.23; 1.46)^a**^	0.94 (0.29; 2.72)	0.93 (0.38; 2.69)	<0.001
GGT (U/l)	17.10 (11.00; 27.33)	13.50 (8.90; 22.90)	16.75 (10.80; 26.50)^b***^	21.90 (14.30; 32.80)^c***^	<0.001
Cotinine (ng/ml)	175.00 (67.15; 291.25)	168.00 (32.48; 284.50)	183.50 (71.15; 286.75)	179.00 (73.93; 310.00)	0.518
Glucose (mmol/l)	5.04 (4.77; 5.30)	4.98 (4.72; 5.26)	5.05 (4.80; 5.29)	5.08 (4.79; 5.34)	0.008
Insulin (µU/l)	8.39 (5.98; 11.89)	7.71 (5.69; 10.41)	8.40 (5.85; 12.45)	9.11 (6.53; 12.94)^c**^	<0.001
HOMA-IR (%)	1.89 (1.31; 2.68)	1.71 (1.23; 2.39)	1.88 (1.29; 2.74)	2.02 (1.44; 2.91)^c***^	<0.001

Data presented as mean (SD) or median (25; 75^th^ percentile).

Notes: values are expressed as arithmetic mean ± standard deviation, geometric mean with 25th and 75th percentiles, or frequency and percentage.

*24h-SBP*, 24-hour Systolic blood pressure; *24h-DBP*, 24-hour Diastolic blood pressure; *24h-PP*, 24-hour pulse pressure; *Office SBP*, Office systolic blood pressure; *Office DBP*, Office diastolic blood pressure; *Office PP*, Office pulse pressure; *clinical MAP*, clinical Mean Arterial Pressure; *SygmoCor MAP*, SygmoCor Mean Arterial Pressure; *PWV*, Pulse wave velocity; *Aix*, Augmentation index; *cIMT*, Carotid-intima media thickness; *Total VSS*, Total Vascular status score; *HDL-C*, high-density lipoprotein cholesterol; *LDL-C*, low-density lipoprotein cholesterol; *GGT*, gamma-glutamyl transferase.

^●^ All Cause hypertensive status includes all phenotypes of hypertension (Sustained hypertension, Masked hypertension and White coat hypertension).

▪ PWV recorded is adjusted for central MAP.

^♦^ % Within tertile.

^◊^ % Whitin total group.

P-values are indicated as: * P < 0.05; ** P < 0.01; ***P < 0.001.

^a^ Comparison between tertile 1 and 2.

^b^ Comparison between tertile 2 and 3.

^c^ Comparison between tertile 1 and 3.

Figure S1 depicts the receiver operation characteristics (ROC) analyses for the VSS and the individual components of the VSS within each BP phenotype. In the all-cause hypertension phenotype, the VSS reported an area under the curve (AUC) of 0.65 (0.30 specificity and 0.51 sensitivity). However, PWV presented with an AUC of 0.7 (0.39 specificity and 0.69 sensitivity), specifically in the all-cause HT group. Aix and cIMT both presented with an AUC of less than 0.6 (specificity and sensitivity less than 0.5), considered a chance finding.

Table 2 presents the adjusted odds ratios of the VSS and its individual components to identify each hypertension phenotype, respectively. Despite having an AUC of 0.65, at a VSS of 4, the odds of MHT (1.98; P = 0.048), SHT (2.35; P = 0.002), WCHT (1.74; P = 0.025), and all-cause HT (2.08; P = 0.038) were significant.

**Table 2:** Odds of having hypertension across phenotypes above the calculated vascular marker cut-points.

Hypertension Phenotypes (ENTER) (ONLY NON-MODIFIABLE FACTORS + LIFESTYLE + BIOMARKERS)												
	All cause hypertension			Masked Hypertension			White-coat hypertension			Sustained hypertension		
	Nagelkerke R^2^: 0.41			Nagelkerke R^2^: 0.40			Nagelkerke R^2^: 0.39			Nagelkerke R^2^: 0.57		
	Adjusted OR	95% CI	P-Value	Adjusted OR	95% CI	P-Value	Adjusted OR	95% CI	P-Value	Adjusted OR	95% CI	P-Value
PWV CP (m/s)	2.70	2.54; 3.02	0.005	2.56	2.31; 2.88	0.019	NS	NS	NS	2.25	2.09; 2.57	0.006
Age (years)	NS	NS	NS	1.75	1.56; 2.01	0.008	NS	NS	NS	NS	NS	NS
Sex	1.56	1.21; 1.85	<0.001	1.35	1.11; 1.51	<0.001	NS	NS	NS	1.35	1.15; 1.64	0.039
Ethnicity	0.68	0.39; 0.91	0.004	0.41	0.27; 0.64	0.028	NS	NS	NS	NS	NS	NS
Waist circumference	1.78	1.48; 1.96	<0.001	2.15	1.84; 2.39	<0.001	NS	NS	NS	3.22	3.05; 3.69	<0.001
SygmoCor MAP	3.51	2.97; 4.02	<0.001	2.16	1.94; 2.41	<0.001	7.05	5.88; 9.03	<0.001	8.63	5.42; 10.55	<0.001
C-Reactive protein	NS	NS	NS	NS	NS	NS	1.58	1.22; 1.98	0.041	1.84	1.48; 2.21	0.002
Total Cholesterol	NS	NS	NS	NS	NS	NS	NS	NS	NS	NS	NS	NS

	All cause hypertension			Masked Hypertension			White-coat hypertension			Sustained hypertension		
	Nagelkerke R^2^: 0.34			Nagelkerke R^2^: 0.28			Nagelkerke R^2^: 0.22			Nagelkerke R^2^: 0.31		
	Adjusted OR	95% CI	P-Value	Adjusted OR	95% CI	P-Value	Adjusted OR	95% CI	P-Value	Adjusted OR	95% CI	P-Value
cIMT CP(mm)	NS	NS	NS	NS	NS	NS	NS	NS	NS	NS	NS	NS
Age (years)	NS	NS	NS	NS	NS	NS	NS	NS	NS	NS	NS	NS
Sex	1.56	1.21; 1.85	<0.001	1.35	1.11; 1.51	<0.001	1.54	1.22; 1.83	0.013	1.35	1.15; 1.64	0.037
Ethnicity	1.56	1.35; 1.74	0.001	1.68	1.26; 2.06	<0.001	NS	NS	NS	NS	NS	NS
Waist circumference	1.88	1.44; 2.15	<0.001	1.98	1.53; 2.41	<0.001	NS	NS	NS	3.05	2.36; 3.97	<0.001
SygmoCor MAP	3.65	2.97; 4.08	<0.001	2.26	1.78; 3.14	<0.001	7.24	5.65; 8.05	<0.001	9.64	7.44; 11.98	<0.001
C-Reactive protein	NS	NS	NS	NS	NS	NS	1.55	1.21; 1.71	0.034	1.15	1.06; 1.34	0.001
Total Cholesterol	NS	NS	NS	NS	NS	NS	NS	NS	NS	NS	NS	NS

	All cause hypertension			Masked Hypertension			White-coat hypertension			Sustained hypertension		
	Nagelkerke R^2^: 0.44			Nagelkerke R^2^: 0.39			Nagelkerke R^2^: 0.26			Nagelkerke R^2^: 0.52		
	Adjusted OR	95% CI	P-Value	Adjusted OR	95% CI	P-Value	Adjusted OR	95% CI	P-Value	Adjusted OR	95% CI	P-Value
Aix CP (%)	2.45	1.84; 3.05	0.006	2.48	1.93; 2.78	0.003	NS	NS	NS	3.12	2.25; 4.08	0.028
Age (years)	NS	NS	NS	1.98	1.68; 2.09	0.016	NS	NS	NS	NS	NS	NS
Sex	1.98	1.35; 2.85	<0.001	2.35	1.65; 3.08	<0.001	2.16	1.62; 2.49	0.048	2.36	1.94; 2.87	0.007
Ethnicity	0.69	0.39; 0.92	0.004	0.42	0.28; 0.64	0.029	NS	NS	NS	NS	NS	NS
Waist circumference	1.97	1.54; 2.15	<0.001	2.05	1.66; 2.49	<0.001	NS	NS	NS	3.15	2.54; 3.99	<0.001
SygmoCor MAP	4.62	3.05; 5.14	<0.001	3.19	2.55; 3.94	<0.001	8.69	6.41; 10.69	<0.001	8.69	6.58; 9.69	<0.001
C-Reactive protein	NS	NS	NS	NS	NS	NS	1.69	1.26; 2.42	0.042	1.76	1.33; 2.08	0.003
Total Cholesterol	NS	NS	NS	NS	NS	NS	NS	NS	NS	NS	NS	NS

	All cause hypertension			Masked Hypertension			White-coat hypertension			Sustained hypertension		
	Nagelkerke R^2^: 0.39			Nagelkerke R^2^: 0.41			Nagelkerke R^2^: 0.39			Nagelkerke R^2^: 0.57		
	Adjusted OR	95% CI	P-Value	Adjusted OR	95% CI	P-Value	Adjusted OR	95% CI	P-Value	Adjusted OR	95% CI	P-Value
VSS CP	2.08	1.69; 2.38	0.038	1.98	1.64; 2.34	0.048	1.74	1.23; 2.05	0.025	2.35	1.97; 2.73	0.002
Age (years)	NS	NS	NS	NS	NS	NS	NS	NS	NS	NS	NS	NS
Sex	1.58	1.18; 2.34	<0.001	1.22	1.11; 1.74	<0.001	1.69	1.28; 2.02	0.014	1.28	1.02; 1.45	0.035
Ethnicity	0.65	0.35; 0.82	0.009	0.46	0.30; 0.71	<0.001	NS	NS	NS	NS	NS	NS
Waist circumference	1.98	1.46; 2.34	<0.001	2.97	2.34; 3.24	<0.001	NS	NS	NS	2.66	1.65; 3.27	<0.001
SygmoCor MAP	3.78	2.59; 4.25	<0.001	3.19	1.94; 4.08	<0.001	7.21	5.98; 8.57	<0.001	8.52	6.41; 9.87	<0.001
C-Reactive protein	NS	NS	NS	NS	NS	NS	1.57	1.24; 1.82	0.031	1.16	1.09; 1.39	0.007
Total Cholesterol	NS	NS	NS	NS	NS	NS	NS	NS	NS	NS	NS	NS

Adjusted for: age, sex, ethnicity, socio-economic status, waist circumference, sygmoCor MAP, self-reported alcohol use, self-reported smoking, CRP and Total cholesterol.

*MAP*, Mean Arterial Pressure; *PWV*, Pulse wave velocity; *Aix*, Augmentation index; *cIMT*, Carotid-intima media thickness; *VSS*, Vascular status score; *HDL-C*, high-density lipoprotein cholesterol; *LDL-C*, low-density lipoprotein cholesterol; *GGT*, gamma-glutamyl transferase.

Yet, at the PWV cut-points, as determined by ROC, (MHT: 6.4 m/s^2^; SHT:6.7 m/s^2^; All cause HT: 6.3 m/s^2^) the odds of MHT (2.56; P = 0.019), SHT (2.25; p = 0.006) and all-cause HT (2.70; P = 0.005) were significantly increased. An AIx above the cut-points (MHT: 4.3%; and SHT: 5.3%; All cause HT: 4.3%) increased the odds of MHT (2.48; P = 0.003), SHT (3.12; P = 0.028) and all-cause HT (2.54; P = 0.006) significantly. Odds ratios in cIMT did not reveal any significant result.

## Discussion

In this observational study, we evaluated the ability of the composite VSS and its constituents, PWV, AIx, and cIMT, in identifying hypertension regardless of phenotype, in a young, apparently healthy South African population. Of these measures, PWV demonstrated superior discriminatory ability across all hypertension phenotypes, outperforming both the composite VSS, AIx and cIMT. These findings support PWV as a sensitive predictor of hypertension in young adults before overt CVD manifestation.

Increased or higher PWV and AIx are well-established markers of arterial stiffness, and both independently associate with HT and elevated CV risk, alongside increased cIMT, which also links with HT and consequently CV risk [37, 38]. Although PWV, Aix, and cIMT each describe a very distinct characteristic of the vasculature, they are not fully independent from one another [19, 39].

PWV quantifies the speed at which the arterial pressure wave travels along elastic arteries and is directly dependent on arterial wall elasticity and compliance [40]. According to the Moens–Korteweg relationship, the propagation velocity increases as the arterial wall stiffens due to reduced distensibility, increased collagen-to-elastin ratio, and early fragmentation of elastic fibres [41]. These changes elevate central pressures and impulse transmission to microcirculatory beds, initiating vascular remodelling that precedes clinically measurable structural thickening observable with cIMT assessment. Consequently, PWV reflects a dynamic, functional index of vascular aging that responds earlier to hemodynamic and molecular alterations than structural indices such as cIMT [42]. AIx on the other hand, represents wave reflection phenomena influenced by heart rate, peripheral vascular resistance, and arterial tone [43, 44]. Although elevated AIx has been associated with hypertension and increased cardiovascular risk, its dependence on transient hemodynamic conditions limits sensitivity in younger persons with compliant arteries and minimal reflection site alterations [45, 46]. Similarly, cIMT primarily captures longer-term structural adaptations of arterial walls to chronic mechanical load and inflammation, which may not yet be apparent in early hypertension [11]. Therefore, integrating these indices into a composite VSS may inadvertently dilute the diagnostic weight of PWV in phenotypically healthy young adults whose predominant vascular alteration is functional stiffening rather than morphological remodelling.

The observed superiority of PWV aligns with prior findings that identify it as the gold standard marker of arterial stiffness and one of the earliest detectable manifestations of vascular dysfunction preceding sustained hypertension and atherosclerosis [47]. Physiologically, small increases in pulse wave velocity within the normal range may reflect early derangements in smooth muscle tone, extracellular matrix composition, and endothelial dysfunction, all contributors to the initiation of elevated systolic pressure and altered pressure wave reflection [48]. These processes are particularly important in MHT and WCHT, where intermittent or stress-induced rises in pressure can accelerate central arterial stiffening before consistent clinical elevation in brachial BP.

Notably, unlike temporary BP fluctuations that may misclassify hypertensive phenotypes, PWV provides an integrated measure of cumulative arterial stress and early load-bearing adaptation [49]. Its reproducibility, non-invasive nature, and feasibility for large-scale implementation further enhance its clinical utility, particularly for identifying high-risk young adults with normal clinic BP readings [50]. However, population-specific reference values for PWV in South African adults remain scarce. While recent paediatric findings in South African children (ages 6–16 years) using the Vicorder device, showed relatively flat PWV trajectories (gender-specific percentiles by age and height), no comprehensive age-stratified normograms are available for young South African adults [51]. Earlier work from the AfricanPredict cohort noted higher PWV in Black South Africans compared to White South Africans, but lacked formal adult reference curves [10]. Our study-derived optimal cut-points in terms of hypertension, thus filling a critical gap, though future research should establish broader normograms to enhance PWV’s clinical interpretability in this population.

The ability of PWV to sensitively detect hypertension phenotypes in young adults, prior to the emergence of clinically overt cardiovascular risk markers, may enable improved risk stratification, potentially reducing long-term cardiovascular risk and morbidity. Indeed, identifying hypertension in young adults is critically important regardless of phenotype as early-onset hypertension substantially increases the risk of developing CVD, organ damage, and mortality later in life, even in the absence of clear clinical markers [52]. Given the often-silent nature of hypertension in young adults, especially in conditions like MHT and WCHT, reliable and sensitive markers are essential for accurate identification.

While composite vascular indices remain conceptually valuable for capturing multidimensional vascular health, the present results suggest that PWV alone provides a more sensitive and physiologically direct measure of early hypertension-related vascular dysfunction in this demographic. Future research should validate these findings in longitudinal cohorts to determine whether progressive increases in PWV predict transitions between hypertension phenotypes and subsequent CVD outcomes. Integration of PWV with emerging functional vascular markers, such as endothelial reactivity or microvascular impedance, may further refine early cardiovascular risk prediction.

PWV may offer a more straightforward, non-invasive application for early hypertension detection in young adults. At the population level, carotid-femoral PWV could be integrated into community screening programs, such as wellness days or university health fairs targeting 20-30-year-olds. At our cut-point of MHT: 6.4 m/s^2^ for MHT, 6.7 m/s^2^ for SHT and all cause HT at 6.3 m/s^2^) the odds of MHT (2.56; P = 0.019), SHT (2.25; P = 0.006) and all-cause HT (2.70; P = 0.005). Especially for MHT and SHT, enabling risk stratification at low cost, using portable tonometers. This outperforms the composite VSS by avoiding multi-measure integration, reducing operator training needs in resource-limited settings like North West Province.

From a more clinical perspective, PWV may fit into primary care workflows, as it may be measured in easily during routine BP checks using devices like the SphygmoCor or Vicorder, validated in African cohorts. Positive screens (those above the cut-point) would prompt 24-hour ambulatory BP monitoring, prioritizing high-risk phenotypes underserved by office BP alone. For instance, in a clinic serving 50 young adults weekly, PWV could identify 8–10 cases of masked hypertension missed by guidelines, facilitating early lifestyle interventions or low-dose therapy per 2024 ESC recommendations. Unlike AIx (requiring additional software applications and transformations) or cIMT (requiring specialized ultrasound), PWV’s simplicity supports scale-up without advanced infrastructure. These applications underscore PWV’s role as a robust, standalone biomarker, enhancing equity in hypertension management for young African populations.

## Strengths and limitations

This non-invasive hypertension risk prediction model for young adults is highly novel, as this is the first study, to our knowledge, to explore the use of the VSS in identifying incident hypertension within a South African cohort. Furthermore, our South African population exhibits different lifestyle characteristics compared to the Swedish population, where the score was originally developed. Our relatively large population size and robust markers, particularly the gold standard for measuring blood pressure and defining hypertension phenotypes (ABPM) and arterial stiffness (PWV), laid the foundation for future studies. While PWV demonstrated superior non-invasive diagnostic utility for identifying hypertension phenotypes in this young adult cohort, its clinical adoption may be tempered by equipment costs and availability, particularly in resource-limited settings like South Africa. Non-invasive carotid-femoral PWV devices, though increasingly accessible, require specialized training and investment, which could limit routine use in primary care. Yet future studies should evaluate cost-effective alternatives or implementation strategies to broaden PWV’s application as a reliable early indicator of hypertension and CVD risk. The cross-sectional study design limits the ability to establish causality, and the findings may not be representative of the entire South African population, as participants were only from a specific region in the North-West Province. Additionally, as the current study is cross-sectional, we cannot infer any clinical recommendations or endorsements for the derived cut-points. However, as the Arican-PREDICT study is an ongoing, longitudinal study, follow-up investigations can verify these findings overtime, and these derived cut-points and observations can be assessed in different populations – as our study only included two ethnic groups within South Africa from a specific region in South Africa, preventing extrapolation. Yet, the external validity of our PWV findings may translate to other young adult populations (aged 20–30) with similar cardiometabolic risk profiles, particularly those undergoing epidemiologic transition like urbanizing African and South Asian groups [53]. Our community-based cohort mirrors demographics in studies like DAWN-Japan (young adults where PWV predicts MHT) [54]. However, generalizability may be moderated by regional factors such as higher baseline PWV in Black Africans vs. White or Asian populations, due to genetic/environmental influences, urban lifestyle and access to healthcare facilities. Our findings are thus highly relevant to sub-Saharan Africa and global diaspora, but validation in more rural/low-SES and/or non-sub-Saharan African cohorts is warranted. Phenotype-specific performance (MHT/SHT) should generalize where ambulatory BP is feasible, as PWV tracks arterial stiffening universally in early adulthood. Future studies should evaluate the efficacy of the VSS in a clinically compromised population, such as individuals with established CVD, where it may demonstrate greater ability to identify structural and or functional abnormalities in the vasculature and stratify CV risk.

## Conclusion

Carotid-femoral pulse wave velocity (PWV) outperformed the composite VSS in identifying undiagnosed HT, regardless of phenotype. PWV is a valuable measure of arterial stiffness to consider when evaluating the vasculature of young adults, given that vascular changes preceding HT may be functional rather than structural. In our population, PWV was a superior vascular stiffness marker compared to AIx, cIMT, or the composite VSS, for the early identification of hypertension phenotypes in young adults. Its physiological basis as a direct measure of arterial stiffness supports its clinical value as a predictive biomarker in young populations, supporting its prioritization in hypertension screening and cardiovascular risk assessment. However, caution is warranted when assuming that composite variables like the VSS will necessarily improve predictive accuracy. Without rigorous validation, composite scores may not outperform their individual components, especially in healthier cohorts.

## Summary Table

### What is known about the topic

- The vascular status score (VSS) combines pulse wave velocity (PWV), augmentation index (AIx), and carotid intima-media thickness (cIMT) to assess vascular changes. The VSS detects subclinical vascular dysfunction in metabolic changes, but its role in hypertension is unknown.
- Hypertension phenotypes (sustained, masked, white-coat) vary in vascular risk, necessitating early biomarkers like PWV (arterial stiffness), AIx (wave reflection), and cIMT (atherosclerosis).
- PWV, AIx, and cIMT independently associate with hypertension and CVD risk, but are interdependent. VSS composite detects subclinical vascular dysfunction preceding metabolic/inflammatory changes, suggesting potential for early hypertension identification.

### What this study adds

- PWV alone outperforms VSS, AIx, and cIMT in sensitivity/specificity for all hypertension phenotypes in young South African adults, as it directly captures early functional stiffening and cumulative arterial stress missed by composites.
- Elevated VSS modestly raises odds, but PWV independently identifies sustained, masked and white-coat hypertension, enabling silent phenotype detection before overt CV damage, highlighting PWV’s clinical utility for risk stratification in young adults, reducing long-term morbidity.
- Suggests prioritizing PWV over composites in young adults, with future longitudinal validation for phenotype transitions and CVD prediction

## Supplementary information

Supplementary Material

Supplementary Material - Tables and Figure legends

## Data Availability

The data analysed during the current study are available from the corresponding author (AW) upon reasonable request.
