# Supplementary material for: When the sum isn’t the whole - The vascular status score and its components in hypertension identification: The African PREDICT study
Source: J Hum Hypertens. 2026 May 9;40(7):523–32. doi: 10.1038/s41371-026-01156-3 (PMC13345915; doi:10.1038/s41371-026-01156-3)
Supplement: Supplementary file 1 — Supplementary Material [file 41371_2026_1156_MOESM1_ESM.docx]

## **SUPPLEMENTARY MATERIAL**

**Supplementary Table 1: Basic comparisons within each ethnic group in African-PREDICT**

|  | **Total Group**  **n= 1020** | **Black**  **n= 475** | | | **P-value** | **White**  **n= 545** | | | **P-value** |
| --- | --- | --- | --- | --- | --- | --- | --- | --- | --- |
|  |  | **VSS Tertile 1**  **n = 149** | **VSS Tertile 2**  **n = 122** | **VSS Tertile 3**  **n = 204** |  | **VSS Tertile 1**  **n = 239** | **VSS Tertile 2**  **n = 117** | **VSS Tertile 3**  **n = 189** |  |
| **Demographic and Lifestyle Parameters** | | | | | | | | | |
| Age (years) | 24.50 ± 3.11 | 23.59 ± 2.96 | 24.53 ± 3.30 | 25.06 ± 3.08^c***^ | <0.001 | 23.65 ± 2.98^a**^ | 24.81 ± 2.77 | 25.50 ± 3.08^c***^ | <0.001 |
| Sex (men, %) | 490 (48.0) | 82 (17.3) | 56 (11.8) | 95 (20.0) | 0.210 | 111 (20.4) | 49 (9.0) | 97 (17.8) | 0.263 |
| Body mass index (Kg/m^2^) | 23.89 (20.96; 27.43) | 21.80 (19.51; 26.07) | 23.34 (20.20; 27.28) | 23.41 (20.64; 27.72) | 0.050 | 23.23 (20.98; 26.56)^a*^ | 27.75 (22.50; 27.31) | 25.80 (22.32; 29.68)^c***^ | <0.001 |
| Waist circumference (cm) | 77.50 (70.5; 86.50) | 73.50 (67.45; 81.10) | 76.10 (70.45; 83.50) | 76.50 (70.53; 83.43) | 0.68 | 78.10 (70.00; 86.50) | 79.00 (73.45; 89.50)^b*^ | 84.60 (73.43; 94.35)^c***^ | <0.001 |
| Alcohol use, n (%) | 569 (55.8) | 84 (17.7) | 58 (12.2) | 119 (25.1) | 0.157 | 116 (21.3) | 71 (13.0) | 121 (22.2) | 0.004 |
| Smoking status, yes n (%) | 236 (23.1) | 46 (9.7) | 24 (5.1) | 49 (10.3) | 0.088 | 52 (9.5) | 21 (3.9) | 44 (8.1) | 0.538 |
| Contraceptive use (female, %) | 226 (22.2) | 27 (5.7) | 23 (4.8) | 50 (10.5) | 0.350 | 51 (9.4) | 32 (5.9) | 43 (7.9) | 0.489 |
| Physical activity (TEE)  n= 828 | (1960.50; 2523.25) | 2110.00 (1928.75; 2303.75) | 2113.00 (1898.00; 2358.00) | 2193.00 (1978.00; 2401.00) | 0.257 | 2234.00 (1944.00; 2549.50) | 2285.00 (2043.50; 2547.00) | 2349.00 (2052.00; 2710.50) | 0.254 |
| *Socio-economic Status Class* | | | | | | | | | |
| Low n (%) | 383 (37.5) | 95 (20.0) | 73 (15.4) | 107 (22.5) | 0.278 | 57 (10.5) | 20 (3.7) | 31 (5.7) | <0.001 |
| Middle n (%) | 306 (30.0) | 35 (7.4) | 33 (7.0) | 65 (13.7) | 0.278 | 87 (16.0) | 43 (7.9) | 43 (7.9) | <0.001 |
| High n (%) | 330 (32.4) | 18 (3.8) | 16 (3.4) | 32 (6.7) | 0.278 | 95 (17.4) | 54 (9.9) | 115 (21.1) | <0.001 |
| **Blood pressure measurements** | | | | | | | | | |
| 24h SBP (mmHg) | 116 (110; 123) | 116 (110; 120) | 113 (109; 121) | 116 (110; 123) | 0.199 | 115 (108; 122) | 117 (111; 126) | 120 (113; 126)^c***^ | <0.001 |
| 24h DBP (mmHg) | 68 (65; 72) | 68 (64; 71) | 68 (64; 71)^b**^ | 70 (65; 74)^c***^ | <0.001 | 67 (63; 70)^a*^ | 69 (65; 72)^b***^ | 71 (67; 75)^c***^ | <0.001 |
| 24h PP (mmHg) | 47 (43; 53) | 47 (44; 52) | 46 (42; 51) | 45 (41; 51) | 0.091 | 49 (43; 54) | 49 (44; 54) | 48 (44; 53) | 0.823 |
| 24h MAP (mmHg) | 88 (83; 92) | 87 (82; 91) | 86 (83; 90) | 88 (84; 93) | 0.005 | 86 (82; 90) | 88 (84; 93) | 90 (85; 95) | <0.001 |
| Office SBP (mmHg) | 118 (110; 127) | 115 (110; 123) | 118 (108; 127)^b*^ | 120 (112; 130)^c**^ | <0.001 | 115 (107; 123) | 117 (109; 127) | 120 (113; 131)^c***^ | <0.001 |
| Office DBP (mmHg) | 78 (74; 83) | 77 (73; 82) | 77 (73; 82)^b***^ | 82 (76; 88)^c***^ | <0.001 | 76 (71; 80)^a*^ | 77 (73; 83)^b*^ | 79 (75; 84)^c***^ | <0.001 |
| Office PP (mmHg) | 39 (33; 45) | 39 (33; 45) | 38 (32; 45) | 38 (33; 43) | 0.473 | 39 (33; 45) | 38 (32; 44) | 39 (33; 46) | 0.436 |
| Clinic MAP (mmHg) | 93 (88; 100) | 92 (87; 98) | 91 (87; 98) | 96 (91; 102) | <0.001 | 92 (86; 96) | 92 (87; 100) | 95 (90; 102) | <0.001 |
| Central MAP (mmHg) | 86 (82; 92) | 86 (82; 91)^a*^ | 87 (84; 93)^b***^ | 92 (87; 98)^c***^ | <0.001 | 81 (77; 86)^a***^ | 86 (80; 90)^b***^ | 89 (84; 95)^c***^ | <0.001 |
| Heart rate (Beats/min) | 64 (57; 71) | 59 (53; 68) | 63 (56; 69) | 63 (57; 72)c*** | 0.001 | 63 (57; 69)^a**^ | 68 (60; 73) | 68 (63; 75)^c***^ | <0.001 |
| *BP Phenotype* | | | | | | | | | |
| Normotensive, n (%) | 775 (76.1) | 130 (87.2) | 99 (81.8) | 143 (70.1) | 0.001 | 191 (79.9) | 85 (72.6) | 127 (67.2) | 0.013 |
| White-coat,  n (%) | 45 (4.4) | 4 (2.7) | 6 (5.0) | 19 (9.3) | 0.001 | 3 (1.3) | 5 (4.3) | 8 (4.2) | 0.013 |
| Masked,  n (%) | 156 (15.3) | 13 (8.7) | 12 (9.9) | 23 (11.3) | 0.001 | 42 (17.6) | 24 (20.5) | 42 (22.2) | 0.013 |
| Sustained hypertensive, n (%) | 43 (4.2) | 2 (1.3) | 4 (3.3) | 19 (9.3 ) | 0.001 | 3 (1.3) | 3 (2.6) | 12 (6.3) | 0.013 |
| All cause Hypertensive status, n (%) | 244 (23.9) | 19 (12.8) | 22 (18.2) | 61 (29.9) | <0.001 | 48 (20.1) | 32 (27.4) | 62 (32.8) | 0.011 |
| **Macrovascular Measurement** | | | | | | | | | |
| PWV (m/s) | 6.3 (5.8; 6.9) | 5.9 (5.7; 6.6)^a*^ | 6.2 (5.8; 6.7)^b***^ | 6.7 (6.2; 7.3)^c***^ | <0.001 | 5.9 (5.5; 6.3)^a***^ | 6.2 (5.9; 6.9) | 6.7 (6.2; 7.1)^c***^ | <0.001 |
| Aix (%) | 4.0 (-4.0; 11.5) | -2.5 (-8.3; 4.5)^a***^ | 4.8 (-0.6; 10.5)^b***^ | 11.3 (6.0; 18.0)^c***^ | <0.001 | -5.0 (-10.0; 1.0)^a***^ | 3.5 (-0.8; 10.0)^b***^ | 10.5 (4.5; 16.5)^c***^ | <0.001 |
| cIMT (mm) | 0.03 (0.02; 0.04) | 0.02 (0.01; 0.03)^a***^ | 0.03 (0.02; 0.03)^b***^ | 0.03 (0.03; 0.04)^c***^ | <0.001 | 0.02 (0.1; 0.03)^a***^ | 0.03 (0.02; 0.03)^b***^ | 0.04 (0.03; 0.04)^c***^ | <0.001 |
| Total VSS, n (%) | 3.0 (2.0; 4.0) | 2.0 (1.0; 2.0) | 3.0 (3.0; 3.0) | 4.0 (4.0; 5.0) | <0.001 | 2.0 (1.0; 2.0) | 3.0 (3.0; 3.0) | 4.0 (4.0; 5.0) | <0.001 |
| **Biochemical markers** | | | | | | | | | |
| Total cholesterol (mmol/l) | 3.66 (2.91; 4.55) | 3.28 (2.63; 3.91) | 3.50 (2.77; 4.15) | 3.52 (2.97; 4.27)^c**^ | 0.004 | 3.46 (2.74; 4.52)^a*^ | 4.17 (3.04; 4.98)^b*^ | 4.53 (3.58; 5.22)^c***^ | <0.001 |
| HDL-C (mmol/l) | 1.12 (0.84; 1.40) | 1.05 (0.81; 1.37) | 1.16 (0.85; 1.38) | 1.18 (0.87; 1.41) | 0.378 | 1.05 (0.79; 1.40) | 1.16 (0.84; 1.47) | 1.13 (0.89; 1.47) | 0.050 |
| LDL-C (mmol/l) | 2.29 (1.74; 3.02) | 2.07 (1.48; 2.67) | 2.11 (1.62; 2.79) | 2.16 (1.68; 2.81)^c*^ | 0.046 | 2.23 (1.67; 2.97) | 2.40 (1.83; 3.22)^b**^ | 2.88 (2.18; 3.61)^c***^ | <0.001 |
| Triglycerides (mmol/l) | 0.69 (0.50; 0.99) | 0.58 (0.45; 0.82) | 0.60 (0.48; 0.80)^b**^ | 0.68 (0.52; 0.95)^c**^ | <0.001 | 0.66 (0.49; 0.97)^a**^ | 0.84 (0.62; 1.15) | 0.90 (0.62; 1.30)^c***^ | <0.001 |
| C-reactive protein (mg/l) | 0.81 (0.29; 2.33) | 0.64 (0.25; 1.59) | 0.90 (0.29; 2.73) | 1.18 (0.42; 3.26)^c*^ | 0.024 | 0.53 (0.20; 1.31) | 0.84 (0.35; 2.29) | 0.96 (0.34; 2.79)^c*^ | 0.010 |
| GGT (U/l) | 17.10 (11.00; 27.33) | 19.25 (13.38; 27.90) | 20.80 (12.98; 33.20) | 22.60 (16.4; 35.70)^c*^ | 0.055 | 11.45 (8.10; 17.00)^a*^ | 14.2 (9.35; 22.05)^b**^ | 17.80 (10.80; 30.58)^c***^ | <0.001 |
| Cotinine (ng/ml) | 175.00 (67.15; 291.25) | 244.00 (86.65; 388.00) | 199.50 (90.70; 289.00) | 173.00 (77.20; 280.00) | 0.195 | 130.00 (38.45; 250.50) | 172.00 (47.85; 280.50) | 208.50 (68.50; 328.25) | 0.037 |
| Glucose | 5.04 (4.77; 5.30) | 4.91 (4.65; 5.15) | 5.04 (4.76; 5.29) | 5.02 (4.71; 5.28) | 0.053 | 5.05 (4.82; 5.30) | 5.06 (4.81; 5.33) | 5.12 (4.90; 5.47) | 0.102 |
| Insulin (µU/l) | 8.39 (5.98; 11.89) | 7.90 (5.31; 12.48) | 9.46 (6.30; 12.04) | 9.80 (6.67; 13.39) | 0.154 | 7.34 (5.58; 9.81) | 8.08 (5.87; 10.80) | 8.82 (6.33; 13.45)^c***^ | <0.001 |
| Homa-IR (%) | 1.89 (1.31; 2.68) | 1.79 (1.12; 2.77) | 2.04 (1.37; 2.66) | 2.12 (1.45; 3.06) | 0.138 | 1.65 (1.17; 2.27) | 1.86 (1.33; 2.52) | 1.99 (1.40; 3.11)^c***^ | <0.001 |

Abbreviations: 24h-SBP, 24-hour Systolic blood pressure; 24h-DBP, 24-hour Diastolic blood pressure; 24h-PP, 24-hour pulse pressure; Office SBP, Office systolic blood pressure; Office DBP, Office diastolic blood pressure; Office PP, Office pulse pressure; PWV, Pulse wave velocity; Aix, Augmentation index; cIMT, Carotid-intima media thickness; Total VSS, Total Vascular status score; HDL-C, high-density lipoprotein cholesterol; LDL-C, low-density lipoprotein cholesterol; GGT, gamma-glutamyl transferase.

Notes: values are expressed as arithmetic mean ± standard deviation, geometric mean with 25th and 75th percentiles, or frequency and percentage.

P-values are indicated as: * P<0.05; ** P<0.01; ***P<0.001

^a^ Comparison between tertile 1 and 2.

^b^ Comparison between tertile 2 and 3.

^c^ Comparison between tertile 1 and 3.

| **Supplementary Table 2A: Additional Odds ratios considering non-modifiable covariates** | | | | | | | | | | | | |
| --- | --- | --- | --- | --- | --- | --- | --- | --- | --- | --- | --- | --- |
| **Hypertension Phenotypes (ENTER) (ONLY NON-MODIFIABLE FACTORS)** | | | | | | | | | | | | |
|  | **All cause hypertension** | | | **Masked Hypertension** | | | **White-coat hypertension** | | | **Sustained hypertension** | | |
|  | Adj R^2^: 0.183 | | | Adj R^2^: 0.177 | | | Adj R^2^: 0.124 | | | Adj R^2^: 0.203 | | |
|  | **Adjusted OR** | **95% CI** | **P-Value** | **Adjusted OR** | **95% CI** | **P-Value** | **Adjusted OR** | **95% CI** | **P-Value** | **Adjusted OR** | **95% CI** | **P-Value** |
| PWV CP (m/s) | 0.432 | 0.306; 0.608 | <0.001 | 0.549 | 0.368; 0.820 | 0.003 | 0.344 | 0.161; 0.734 | 0.006 | 0.145 | 0.054; 0.390 | <0.001 |
| Age (years) | 1.051 | 0.895; 1.233 | 0.546 | 0.879 | 0.725; 1.067 | 0.192 | 1.388 | 1.002; 1.924 | 0.049 | 1.628 | 1.145; 2.317 | 0.007 |
| Sex | 0.275 | 0.194; 0.389 | <0.001 | 0.229 | 0.149; 0.350 | <0.001 | 0.405 | 0.198; 0.829 | 0.013 | 0.372 | 0.173; 0.802 | 0.012 |
| Ethnicity | 0.679 | 0.496; 0.929 | 0.016 | 0.412 | 0.279; 0.609 | <0.001 | 1.716 | 0.901; 3.270 | 0.101 | 1.255 | 0.654; 2.409 | 0.495 |
|  | | | | | | | | | | | | |
|  | **All cause hypertension** | | | **Masked Hypertension** | | | **White-coat hypertension** | | | **Sustained hypertension** | | |
|  | Adj R^2^: 0.156 | | | Adj R^2^: 0.166 | | | Adj R^2^: 0.096 | | | Adj R^2^: 0.138 | | |
|  | **Adjusted OR** | **95% CI** | **P-Value** | **Adjusted OR** | **95% CI** | **P-Value** | **Adjusted OR** | **95% CI** | **P-Value** | **Adjusted OR** | **95% CI** | **P-Value** |
| cIMT CP(mm) | 0.779 | 0.572; 1.059 | 0.111 | 0.768 | 0.532; 1.109 | 0.160 | 0.945 | 0.509; 1.755 | 0.859 | 0.687 | 0.357; 1.323 | 0.261 |
| Age (years) | 1.128 | 0.966; 1.317 | 0.128 | 0.921 | 0.763; 1.112 | 0.392 | 1.503 | 1.094; 2.064 | 0.012 | 1.788 | 1.275; 2.508 | <0.001 |
| Sex | 0.208 | 0.149; 0.288 | <0.001 | 0.189 | 0.126; 0.284 | <0.001 | 0.272 | 0.140; 0.530 | <0.001 | 0.207 | 0.100; 0.428 | <0.001 |
| Ethnicity | 0.726 | 0.533; 0.988 | 0.041 | 0.435 | 0.296; 0.640 | <0.001 | 1.886 | 0.996; 3.572 | 0.051 | 1.373 | 0.723; 2.610 | 0.333 |
|  | | | | | | | | | | | | |
|  | **All cause hypertension** | | | **Masked Hypertension** | | | **White-coat hypertension** | | | **Sustained hypertension** | | |
|  | Adj R^2^: 0.171 | | | Adj R^2^: 0.173 | | | Adj R^2^: 0.115 | | | Adj R^2^: 0.154 | | |
|  | **Adjusted OR** | **95% CI** | **P-Value** | **Adjusted OR** | **95% CI** | **P-Value** | **Adjusted OR** | **95% CI** | **P-Value** | **Adjusted OR** | **95% CI** | **P-Value** |
| Aix CP (%) | 0.535 | 0.385; 0.743 | <0.001 | 0.609 | 0.414; 0.897 | 0.012 | 0.451 | 0.232; 0.875 | 0.019 | 0.437 | 0.222; 0.860 | 0.016 |
| Age (years) | 1.087 | 0.929; 1.272 | 0.299 | 0.898 | 0.742; 1.087 | 0.270 | 1.424 | 1.033; 1.963 | 0.031 | 1.765 | 1.255; 2.482 | 0.001 |
| Sex | 0.170 | 0.120; 0.241 | <0.001 | 0.163 | 0.107; 0.248 | <0.001 | 0.216 | 0.108; 0.432 | <0.001 | 0.158 | 0.074; 0.336 | <0.001 |
| Ethnicity | 0.690 | 0.505; 0.942 | 0.019 | 0.422 | 0.286; 0.622 | <0.001 | 1.729 | 0.908; 3.291 | 0.096 | 1.262 | 0.660; 2.414 | 0.482 |
|  | | | | | | | | | | | | |
|  | **All cause hypertension** | | | **Masked Hypertension** | | | **White-coat hypertension** | | | **Sustained hypertension** | | |
|  | Adj R^2^: 0.182 | | | Adj R^2^: 0.175 | | | Adj R^2^: 0.135 | | | Adj R^2^: 0.154 | | |
|  | **Adjusted OR** | **95% CI** | **P-Value** | **Adjusted OR** | **95% CI** | **P-Value** | **Adjusted OR** | **95% CI** | **P-Value** | **Adjusted OR** | **95% CI** | **P-Value** |
| VSS CP | 0.413 | 0.283; 0.602 | <0.001 | 0.561 | 0.370; 0.852 | 0.007 | 0.215 | 0.075; 0.616 | 0.004 | 0.437 | 0.222; 0.860 | 0.016 |
| Age (years) | 1.048 | 0.894; 1.229 | 0.562 | 0.882 | 0.728; 1.069 | 0.201 | 1.366 | 0.988; 1.888 | 0.059 | 1.765 | 1.255; 2.482 | 0.001 |
| Sex | 0.214 | 0.154; 0.233 | <0.001 | 0.193 | 0.129; 0.290 | <0.001 | 0.289 | 0.148; 0.564 | <0.001 | 0.158 | 0.074; 0.336 | <0.001 |
| Ethnicity | 0.670 | 0.490; 0.917 | 0.012 | 0.414 | 0.281; 0.611 | <0.001 | 1.709 | 0.897; 3.257 | 0.103 | 1.262 | 0.660; 2.414 | 0.482 |

Adjust for: age, sex and ethnicity.

Abbreviations: SES, Socio-economic status; Wc, Waist Circumference; Mean Arterial Pressure; PWV, Pulse wave velocity; Aix, Augmentation index; cIMT, Carotid-intima media thickness; Total VSS, Total Vascular status score; HDL-C, high-density lipoprotein cholesterol; LDL-C, low-density lipoprotein cholesterol; GGT, gamma-glutamyl transferase.

| **Supplementary Table 2B: Additional Odds ratios considering non-modifiable and lifestyle covariates** | | | | | | | | | | | | |
| --- | --- | --- | --- | --- | --- | --- | --- | --- | --- | --- | --- | --- |
| **Hypertension Phenotypes (ENTER) (ONLY NON MODIFIABLE FACTORS + LIFESTYLE)** | | | | | | | | | | | | |
|  | **All cause hypertension** | | | **Masked Hypertension** | | | **White-coat hypertension** | | | **Sustained hypertension** | | |
|  | Adj R^2^: 0.403 | | | Adj R^2^: 0.322 | | | Adj R^2^: 0.378 | | | Adj R^2^: 0.541 | | |
|  | **Adjusted OR** | **95% CI** | **P-Value** | **Adjusted OR** | **95% CI** | **P-Value** | **Adjusted OR** | **95% CI** | **P-Value** | **Adjusted OR** | **95% CI** | **P-Value** |
| PWV CP (m/s) | 0.764 | 0.460; 1.016 | 0.060 | 0.720 | 0.462; 1.124 | 0.148 | 0.558 | 0.234; 1.327 | 0.187 | 0.229 | 0.067; 0.775 | 0.018 |
| Age (years) | 0.828 | 0.674; 1.018 | 0.074 | 0.663 | 0.519; 0.848 | 0.001 | 1.176 | 0.797; 1.736 | 0.414 | 1.372 | 0.845; 2.228 | 0.201 |
| Sex | 0.220 | 0.145; 0.336 | <0.001 | 0.255 | 0.158; 0.411 | <0.001 | 0.302 | 0.128; 0.713 | 0.006 | 0.123 | 0.041; 0.375 | <0.001 |
| Ethnicity | 0.579 | 0.370; 0.908 | 0.017 | 0.549 | 0.335; 0.900 | 0.017 | 0.848 | 0.330; 2.179 | 0.732 | 0.764 | 0.250; 2.334 | 0.637 |
| SES | 1.054 | 0.838; 1.325 | 0.655 | 1.234 | 0.952; 1.600 | 0.112 | 0.838 | 0.529; 1.326 | 0.450 | 1.048 | 0.617; 1.779 | 0.863 |
| Waist circumference | 1.704 | 1.401; 2.072 | <0.001 | 1.905 | 1.528; 2.376 | <0.001 | 1.248 | 0.816; 1.907 | 0.306 | 1.757 | 1.099; 2.809 | 0.019 |
| SygmoCor MAP | 3.071 | 2.460; 3.834 | <0.001 | 2.114 | 1.656; 2.699 | <0.001 | 5.572 | 3.470; 8.948 | <0.001 | 7.780 | 4.407; 13.734 | <0.001 |
| Self-reported alcohol | 0.922 | 0.642; 1.325 | 0.662 | 0.820 | 0.542; 1.240 | 0.346 | 1.169 | 0.553; 2.471 | 0.683 | 1.378 | 0.596; 3.185 | 0.454 |
| Self-reported Smoking | 1.421 | 0.927; 2.179 | 0.107 | 1.495 | 0.914; 2.444 | 0.109 | 1.018 | 0.430; 2.411 | 0.967 | 1.540 | 0.558; 4.252 | 0.404 |
|  | | | | | | | | | | | | |
|  | **All cause hypertension** | | | **Masked Hypertension** | | | **White-coat hypertension** | | | **Sustained hypertension** | | |
|  | Adj R^2^: 0.403 | | | Adj R^2^: 0.324 | | | Adj R^2^: 0.373 | | | Adj R^2^: 0.524 | | |
|  | **Adjusted OR** | **95% CI** | **P-Value** | **Adjusted OR** | **95% CI** | **P-Value** | **Adjusted OR** | **95% CI** | **P-Value** | **Adjusted OR** | **95% CI** | **P-Value** |
| cIMT CP(mm) | 0.714 | 0.500; 1.021 | 0.065 | 0.703 | 0.469; 1.053 | 0.087 | 0.881 | 0.428; 1.811 | 0.730 | 0.622 | 0.275; 1.407 | 0.255 |
| Age (years) | 0.838 | 0.683; 1.029 | 0.091 | 0.667 | 0.523; 0.850 | 0.001 | 1.222 | 0.833; 0.106 | 0.305 | 1.424 | 0.892; 2.274 | 0.139 |
| Sex | 0.193 | 0.130; 0.287 | <0.001 | 0.228 | 0.145; 0.359 | <0.001 | 0.238 | 0.106; 0.532 | <0.001 | 0.085 | 0.030; 0.247 | <0.001 |
| Ethnicity | 0.559 | 0.355; 0.879 | 0.012 | 0.543 | 0.330; 0.891 | 0.016 | 0.816 | 0.319; 2.088 | 0.672 | 0.587 | 0.196; 1.755 | 0.341 |
| SES | 1.041 | 0.828; 1.309 | 0.732 | 1.231 | 0.949; 1.596 | 0.117 | 0.816 | 0.518; 1.286 | 0.381 | 0.980 | 0.577; 1.666 | 0.940 |
| Waist circumference | 1.697 | 1.395; 2.063 | <0.001 | 1.895 | 1.521; 2.361 | <0.001 | 1.212 | 0.797; 1.841 | 0.368 | 1.688 | 1.064; 2.679 | 0.026 |
| SygmoCor MAP | 3.235 | 2.599; 4.027 | <0.001 | 2.189 | 1.729; 2.794 | <0.001 | 5.859 | 3.652; 9.397 | <0.001 | 9.039 | 5.080; 16.085 | <0.001 |
| Self-reported alcohol | 0.899 | 0.626; 1.291 | 0.564 | 0.788 | 0.520; 1.192 | 0.258 | 1.153 | 0.544; 2.445 | 0.710 | 1.290 | 0.563; 2.958 | 0.547 |
| Self-reported Smoking | 1.382 | 0.903; 2.116 | 0.137 | 1.444 | 0.885; 2.355 | 0.141 | 1.044 | 0.440; 2.475 | 0.923 | 1.630 | 0.594; 4.473 | 0.342 |
|  | | | | | | | | | | | | |
|  | **All cause hypertension** | | | **Masked Hypertension** | | | **White-coat hypertension** | | | **Sustained hypertension** | | |
|  | Adj R^2^: 0.403 | | | Adj R^2^: 0.323 | | | Adj R^2^: 0.378 | | | Adj R^2^: 0.537 | | |
|  | **Adjusted OR** | **95% CI** | **P-Value** | **Adjusted OR** | **95% CI** | **P-Value** | **Adjusted OR** | **95% CI** | **P-Value** | **Adjusted OR** | **95% CI** | **P-Value** |
| Aix CP (%) | 1.489 | 0.984; 2.254 | 0.060 | 1.455 | 0.907; 2.334 | 0.120 | 1.767 | 0.758; 4.116 | 0.187 | 3.213 | 1.198; 8.613 | 0.020 |
| Age (years) | 0.872 | 0.711; 1.068 | 0.186 | 0.697 | 0.548; 0.886 | 0.003 | 1.251 | 0.856;P 1.829 | 0.247 | 1.406 | 0.880; 2.247 | 0.154 |
| Sex | 0.219 | 0.144; 0.333 | <0.001 | 0.253 | 0.158; 0.405 | <0.001 | 0.285 | 0.123; 0.659 | 0.003 | 0.111 | 0.037; 0.335 | <0.001 |
| Ethnicity | 0.587 | 0.375; 0.920 | 0.020 | 0.552 | 0.337; 0.905 | 0.019 | 0.821 | 0.322; 2.095 | 0.680 | 0.657 | 0.221; 1.953 | 0.450 |
| SES | 1.063 | 0.846; 1.335 | 0.602 | 1.237 | 0.955; 1.601 | 0.107 | 0.841 | 0.533; 1.326 | 0.456 | 1.072 | 0.634; 1.814 | 0.796 |
| Waist circumference | 1.717 | 1.411; 2.090 | <0.001 | 1.922 | 1.539; 2.399 | <0.001 | 1.215 | 0.798; 1.849 | 0.364 | 1.758 | 1.107; 2.796 | 0.017 |
| SygmoCor MAP | 3.462 | 2.739; 4.376 | <0.001 | 2.373 | 1.827; 3.083 | <0.001 | 6.598 | 3.945; 11.032 | <0.001 | 11.687 | 6.138; 22.254 | <0.001 |
| Self-reported alcohol | 0.886 | 0.617; 1.274 | 0.515 | 0.784 | 0.518; 1.186 | 0.249 | 1.105 | 0.522; 2.341 | 0.794 | 1.188 | 0.515; 2.741 | 0.687 |
| Self-reported Smoking | 1.389 | 0.907; 2.127 | 0.131 | 1.465 | 0.899; 2.387 | 0.126 | 0.967 | 0.407; 2.295 | 0.939 | 1.568 | 0.566; 4.339 | 0.387 |
|  | | | | | | | | | | | | |
|  | **All cause hypertension** | | | **Masked Hypertension** | | | **White-coat hypertension** | | | **Sustained hypertension** | | |
|  | Adj R^2^: 0.400 | | | Adj R^2^: 0.319 | | | Adj R^2^: 0.373 | | | Adj R^2^: 0.523 | | |
|  | **Adjusted OR** | **95% CI** | **P-Value** | **Adjusted OR** | **95% CI** | **P-Value** | **Adjusted OR** | **95% CI** | **P-Value** | **Adjusted OR** | **95% CI** | **P-Value** |
| VSS CP | 1.032 | 0.658; 1.619 | 0.891 | 1.082 | 0.668; 1.752 | 0.749 | 0.837 | 0.263; 2.664 | 0.763 | 0.357 | 0.044; 2.907 | 0.336 |
| Age (years) | 0.861 | 0.701; 1.056 | 0.151 | 0.690 | 0.542; 0.880 | 0.003 | 1.222 | 0.832; 1.795 | 0.307 | 1.435 | 0.897; 2.295 | 0.132 |
| Sex | 0.192 | 0.129; 0.286 | <0.001 | 0.226 | 0.144; 0.355 | <0.001 | 0.242 | 0.109; 0.539 | <0.001 | 0.091 | 0.032; 0.259 | <0.001 |
| Ethnicity | 0.579 | 0.370; 0.907 | 0.017 | 0.551 | 0.336; 0.902 | 0.018 | 0.818 | 0.321; 2.089 | 0.675 | 0.625 | 0.212; 1.841 | 0.393 |
| SES | 1.052 | 0.838; 1.322 | 0.661 | 1.232 | 0.951; 1.596 | 0.114 | 0.815 | 0.517; 1.285 | 0.379 | 0.968 | 0.571; 1.639 | 0.902 |
| Waist circumference | 1.685 | 1.387; 2.048 | <0.001 | 1.879 | 1.509; 2.341 | <0.001 | 1.208 | 0.796; 1.835 | 0.374 | 1.625 | 1.031; 2.563 | 0.037 |
| SygmoCor MAP | 3.225 | 2.565; 4.054 | <0.001 | 2.222 | 1.725; 2.862 | <0.001 | 5.717 | 3.490; 9.364 | <0.001 | 8.020 | 4.487; 14.335 | <0.001 |
| Self-reported alcohol | 0.906 | 0.631; 1.300 | 0.591 | 0.803 | 0.531; 1.212 | 0.296 | 1.139 | 0.539; 2.405 | 0.733 | 1.292 | 0.566; 2.950 | 0.544 |
| Self-reported Smoking | 1.392 | 0.908; 2.133 | 0.129 | 1.450 | 0.888; 2.366 | 0.137 | 1.044 | 0.440; 2.478 | 0.921 | 1.620 | 0.594; 4.415 | 0.346 |

**Adjust for: age, sex, ethnicity, SES, wc, sygmoCor MAP, self-reported alcohol use and self-reported Smoking.**

**
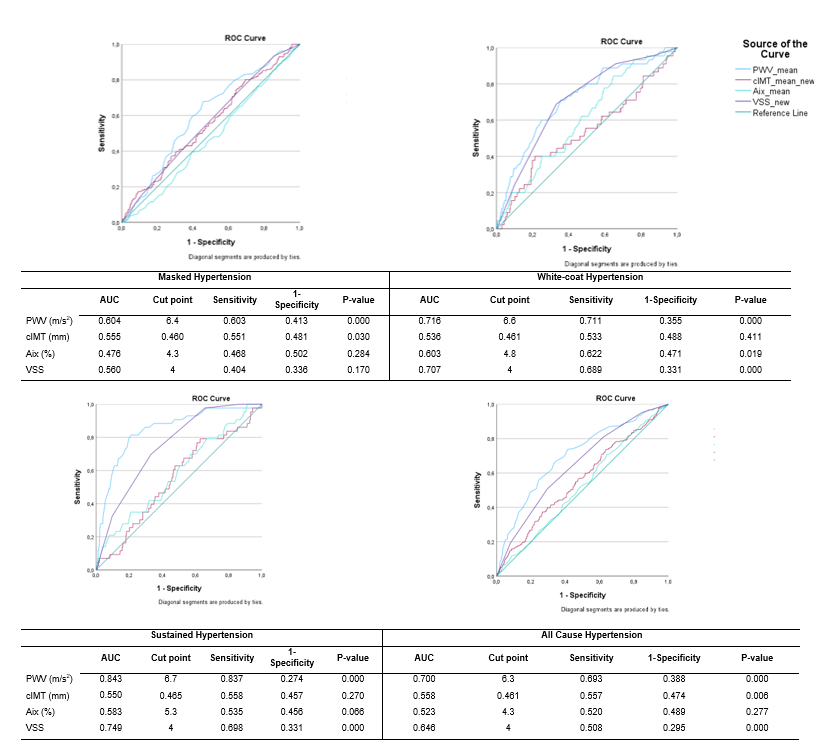
Supplementary Figure – Receiver Operated Characteristics (ROC) curves determining the optimal cutpoint at which PWV, AIx, cIMT and the combined VSS identifies (A) Masked hypertension; (B) White coat Hypertension; (C) Sustained Hypertension and (D) All-cause hypertension.**

**A**

**D**

**C**

**B**

Abbreviations: AIx, augmentation index; AUC, area under the curce; CP, cut-point; cIMT, carotid media intima thickness; PWV, pulse wave velocity; VSS< vascular status score.
