# Supplementary material for: When the sum isn’t the whole - The vascular status score and its components in hypertension identification: The African PREDICT study
Source: J Hum Hypertens. 2026 May 9;40(7):523–32. doi: 10.1038/s41371-026-01156-3 (PMC13345915; doi:10.1038/s41371-026-01156-3)
Supplement: Supplementary file 2 — Supplementary Material - Tables and Figure legends [file 41371_2026_1156_MOESM2_ESM.docx]

## **SUPPLEMENTARY MATERIAL - LEGENDS**

**Supplementary Table 1: Basic comparisons within each ethnic group in African-PREDICT**

Abbreviations: 24h-SBP, 24-hour Systolic blood pressure; 24h-DBP, 24-hour Diastolic blood pressure; 24h-PP, 24-hour pulse pressure; Office SBP, Office systolic blood pressure; Office DBP, Office diastolic blood pressure; Office PP, Office pulse pressure; PWV, Pulse wave velocity; Aix, Augmentation index; cIMT, Carotid-intima media thickness; Total VSS, Total Vascular status score; HDL-C, high-density lipoprotein cholesterol; LDL-C, low-density lipoprotein cholesterol; GGT, gamma-glutamyl transferase.

Notes: values are expressed as arithmetic mean ± standard deviation, geometric mean with 25th and 75th percentiles, or frequency and percentage.

P-values are indicated as: * P<0.05; ** P<0.01; ***P<0.001

^a^ Comparison between tertile 1 and 2.

^b^ Comparison between tertile 2 and 3.

^c^ Comparison between tertile 1 and 3.

**Supplementary Table 2A – Additional Odds Ratios considering non-modifiable covariates**

**Supplementary Table 2B – Additional Odds Ratios considering non-modifiable and lifestyle covariates**

Adjust for: age, sex and ethnicity. Abbreviations: SES, Socio-economic status; Wc, Waist Circumference; Mean Arterial Pressure; PWV, Pulse wave velocity; Aix, Augmentation index; cIMT, Carotid-intima media thickness; Total VSS, Total Vascular status score; HDL-C, high-density lipoprotein cholesterol; LDL-C, low-density lipoprotein cholesterol; GGT, gamma-glutamyl transferase.

**Supplementary Figure – Receiver Operated Characteristics (ROC) curves determining the optimal cutpoint at which PWV, AIx, cIMT and the combined VSS identifies (A) Masked hypertension; (B) White coat Hypertension; (C) Sustained Hypertension and (D) All-cause hypertension.**

Abbreviations: AIx, augmentation index; AUC, area under the curce; CP, cut-point; cIMT, carotid media intima thickness; PWV, pulse wave velocity; VSS< vascular status score.
